# ^31^P magnetic resonance spectroscopy in skeletal muscle: Experts' consensus recommendations

**DOI:** 10.1002/nbm.4246

**Published:** 2020-05

**Authors:** Martin Meyerspeer, Chris Boesch, Donnie Cameron, Monika Dezortová, Sean C. Forbes, Arend Heerschap, Jeroen A.L. Jeneson, Hermien E. Kan, Jane Kent, Gwenaël Layec, Jeanine J. Prompers, Harmen Reyngoudt, Alison Sleigh, Ladislav Valkovič, Graham J. Kemp

**Affiliations:** 1 Center for Medical Physics and Biomedical Engineering, Medical University of Vienna, Vienna, Austria; 2 High Field MR Center, Medical University of Vienna, Vienna, Austria; 3 DBMR and DIPR, University and Inselspital, Bern, Switzerland; 4 Norwich Medical School, University of East Anglia, Norwich, UK; 5 C. J. Gorter Center for High Field MRI, Department of Radiology, Leiden University Medical Centre, Leiden, the Netherlands; 6 MR-Unit, Department of Diagnostic and Interventional Radiology, Institute for Clinical and Experimental Medicine, Prague, Czech Republic; 7 Department of Physical Therapy, University of Florida, Gainesville, Florida, USA; 8 Department of Radiology and Nuclear Medicine, Radboud University Medical Center, Nijmegen, The Netherlands; 9 Department of Radiology, Amsterdam University Medical Center|site AMC, Amsterdam, the Netherlands; 10 Cognitive Neuroscience Center, University Medical Center Groningen, Groningen, the Netherlands; 11 Center for Child Development and Exercise, Wilhelmina Children's Hospital, University Medical Center Utrecht, Utrecht, the Netherlands; 12 Duchenne Center, The Netherlands; 13 Department of Kinesiology, University of Massachusetts Amherst, MA, USA; 14 Institute for Applied Life Sciences, University of Massachusetts, Amherst, MA, USA; 15 Department of Radiology, University Medical Center Utrecht, the Netherlands; 16 NMR Laboratory, Neuromuscular Investigation Center, Institute of Myology AIM-CEA, Paris, France; 17 Wolfson Brain Imaging Centre, University of Cambridge, Cambridge, UK; 18 Wellcome Trust-MRC Institute of Metabolic Science, University of Cambridge, Cambridge, UK; 19 NIHR/Wellcome Trust Clinical Research Facility, Cambridge University Hospitals NHS Foundation Trust, Cambridge, UK; 20 Oxford Centre for Clinical Magnetic Resonance Research (OCMR), RDM Cardiovascular Medicine, BHF Centre of Research Excellence, University of Oxford, Oxford, UK; 21 Department of Imaging Methods, Institute of Measurement Science, Slovak Academy of Sciences, Bratislava, Slovakia; 22 Department of Musculoskeletal Biology and Liverpool Magnetic Resonance Imaging Centre (LiMRIC), University of Liverpool, Liverpool, UK

**Keywords:** ^31^P, exercise, metabolism, MRI, muscle, nuclear magnetic resonance spectroscopy, phosphorus MRS

## Abstract

Skeletal muscle phosphorus-31 (^31^P) MRS is the oldest MRS methodology to be applied to *in vivo* metabolic research. The technical requirements of ^31^P MRS in skeletal muscle depend on the research question, and to assess those questions requires understanding both the relevant muscle physiology, and how ^31^P MRS methods can probe it. Here we consider basic signal-acquisition parameters related to radio frequency excitation, *TR, TE*, spectral resolution, shim and localisation. We make specific recommendations for studies of resting and exercising muscle, including magnetisation transfer, and for data processing. We summarise the metabolic information that can be quantitatively assessed with ^31^P MRS, either measured directly or derived by calculations that depend on particular metabolic models, and we give advice on potential problems of interpretation. We give expected values and tolerable ranges for some measured quantities, and minimum requirements for reporting acquisition parameters and experimental results in publications. Reliable examination depends on a reproducible setup, standardised preconditioning of the subject, and careful control of potential difficulties, and we summarise some important considerations and potential confounders. Our recommendations include the quantification and standardisation of contraction intensity, and how best to account for heterogeneous muscle recruitment. We highlight some pitfalls in the assessment of mitochondrial function by analysis of phosphocreatine (PCr) recovery kinetics. Finally, we outline how complementary techniques (near-infrared spectroscopy, arterial spin labelling, BOLD and various other MRI and ^1^H MRS measurements) can help in the physiological/metabolic interpretation of ^31^P MRS studies by providing information about blood flow and oxygen delivery/utilisation. Our recommendations will assist in achieving the fullest possible reliable picture of muscle physiology and pathophysiology.

## 1 Introduction and Physiological (Metabolic) Background

^31^P MRS studies of skeletal muscle were among the first reported MRS studies of a mammalian organ *in situ*, and in four decades at least 500 such studies of human muscle have been published, more than of any other organ.^1^ MRS methods avoid serious limitations of the classical method for investigating cellular energetics in human skeletal muscle, namely biopsy; these include technical challenges of biochemical analysis (notably delayed metabolic arrest and the instability of high-energy phosphates, especially PCr, in samples before freezing/deproteination), difficulty of data acquisition during exercise (especially multiple measurements in kinetic studies), and limited acceptability, particularly for patients, in repeated or serial studies. Muscles can be studied in various functional states, from the resting state to full contractile activation (using voluntary exercise or electrical stimulation) and during post-exercise metabolic recovery, and in various experimental manipulations such as hypoxia and hyperoxia. *In vivo* ^31^P MRS can detect only free phosphorus-containing metabolites in tissue concentrations of ~100 μM and above, but these include key participants in ATP metabolism and the cellular functions it supports, notably mechanical force production. Here some brief physiological background will set the scene for the main subject of this consensus article, namely technical recommendations on ^31^P MRS muscle experiments and their interpretation.

Mammalian skeletal muscles are composed of multiple muscle cell types (‘myofibres’), of which there are three phenotypically distinct types functionally classified by their contractile and metabolic properties: slow-twitch oxidative (SO), fast-twitch oxidative glycolytic (FOG) and fast-twitch glycolytic (FG) myofibres,^2^ also known on the basis of their different expression of myosin motor proteins as Type I, Type IIa and Type IIb/x respectively. Metabolically, SO fibres are better equipped to oxidise fat and FG fibres to metabolise glucose and glycogen anaerobically to lactate (although they usually work aerobically, generating pyruvate), while FOG fibres are metabolically intermediate.^3^ Under normoxic conditions the mitochondrial reticulum is the main generator of the ATP that provides the energy for fibre contraction and relaxation^4^; the energy available for work is measured by the strongly negative (i.e. far from thermodynamic equilibrium) cytosolic Gibbs free energy of ATP hydrolysis (Δ*G*_ATP_), which reflects a high ATP/ADP concentration ratio (~400 at rest). The contribution of anaerobic glycolytic adenosine diphosphate (ADP) phosphorylation* in resting normoxic skeletal muscle is negligible, but can far exceed mitochondrial ADP phosphorylation,^5^ particularly during high duty cycle, high power contractions.^6^ Myofibres are organised in phenotypically homogeneous clusters innervated by individual somatic neurons (‘motor units’), which are sequentially, not synchronously, recruited during voluntary exercise in a fixed order (SO → FOG → FG motor units) to produce mechanical force.^3^ This underlies the well-known metabolic shift from fat to carbohydrate oxidation during progressive exercise. It also complicates analysis and interpretation of *in vivo* ^31^P MRS muscle recordings in voluntary exercise at submaximal workloads, though this can be somewhat clarified by computational model-based analysis^7^ or alternative experimental strategies such as low-duty-cycle ballistic contractions^8^ or electrical stimulation.^9^

Skeletal muscle is a convenient experimental model to study the ATP synthetic function of the mitochondrial network *in situ*, as it allows exercise studies^†^ in which the metabolic load is manipulated via voluntary or electrically-stimulated contraction. Such dynamic ^31^P MRS exercise-recovery studies have contributed to understanding *in vivo* kinetic control of oxidative ADP phosphorylation in muscle.^1^ In ‘purely oxidative’ exercise (i.e. at moderate workloads below the mechanical threshold of FG motor unit recruitment) under steady-state conditions, mechanical work rate can be used as a surrogate for oxidative ADP phosphorylation rate, and its relationship to metabolic control signals such as free [ADP] or Δ*G*_ATP_ (see Table 1) can be used^10–13^ to make inferences about the muscle's capacity for oxidative ADP phosphorylation.^14^ This interpretation critically depends on localised ^31^P MRS signal collection in the active muscles only, and on accurate quantification of mechanical work. A more robust strategy, relatively independent of workload, is to study the kinetics of PCr resynthesis immediately following moderate exercise. The different technical and interpretative approaches are reviewed elsewhere,^14^ but the idea is that because PCr recovery is almost wholly fuelled by oxidative ATP synthesis, its kinetics reflect muscle ‘mitochondrial capacity’ (sometimes called *Q*_max_), which can be conceptualised as the inferred maximum rate of oxidative ADP phosphorylation under ‘maximum’ stimulation by ^31^P MRS-measurable negative feedback control signals such as [ADP] (although clearly stimulation by other factors, not measurable by ^31^P MRS, such as cytosolic Ca^2+^ or redox state will not be maximal during submaximal exercise).

Another long-standing theme in skeletal muscle physiology is to understand how chemical energy is transformed into mechanical force and power, how this process is controlled,^15^ and how it breaks down at high-contraction duty cycles (muscle fatigue).^16^ *In vivo* ^31^P MRS has made important contributions by correlating mechanical function with the calculated free intramuscular concentrations of ATP, ADP, Pi, Mg^2+^ and H^+^.^16–19^ Also, *in vivo* ^31^P MRS can quantify contractile efficiency,^20^ as the ratio of muscle power or force output (normalised to muscle volume or cross-sectional area) to the total ADP phosphorylation rate, determined from dynamic ^31^P MRS measurements during electrical stimulation or voluntary exercise. This is most straightforwardly done by measuring the initial rate of PCr depletion,^14,20^ although ways are described to estimate the relative contributions of the different ADP phosphorylation pathways, *viz.* the creatine kinase reaction, glycogenolysis and oxidative phosphorylation, as they evolve during exercise.^21^

Exercise studies with ^31^P MRS have also contributed to understanding the control of glycolysis in muscle *in vivo.* ^22–25^ This is most straightforward during exercise under conditions of cuff ischaemia, where glycogenolytic ADP phosphorylation can be estimated from pH and PCr changes in a closed system where oxidative ADP phosphorylation and acid efflux are negligible.^5,26^ Some stoichiometric technicalities of the cellular metabolic production, consumption and buffering of acid (‘H^+^’ in shorthand form) are reviewed elsewhere.^27,28^

## 2 Recommendations for ^31^P MRS Methods

### 2.1 Introduction to the recommendations

Different scientific questions require particular experimental setups and focus on different metabolites, which imposes specific requirements for data quality, such as signal-to-noise ratio (SNR), linewidth, temporal resolution and extent of localisation. The MRS methodology must therefore be tailored to the specific application, while respecting constraints imposed by the instrumentation. SNR depends on, *inter alia*, field strength, coil sensitivity, size and location of the volume of interest (VOI) or voxel—namely, its distance from the coil element(s)—and the linewidth. The latter is, in turn, influenced by shim, and also size and location of the VOI. We make recommendations on signal acquisition for studies of resting muscle (with and without magnetisation transfer) and dynamic studies of muscle exercise. We discuss post-processing steps (fitting, quantifying and deriving physiological parameters from time series). We recommend units for reporting the results, and give some typical values expected in healthy subjects and patients. An overview of the most important recommendations is given at the end of this article. This brief summary can only highlight some important methodical aspects of ^31^P MRS and subject preparation but cannot go into depth and does not cover aspects of interpreting the data.

### 2.2 Signal acquisition

#### 2.2.1 General features of acquisition

**Figure 1:**
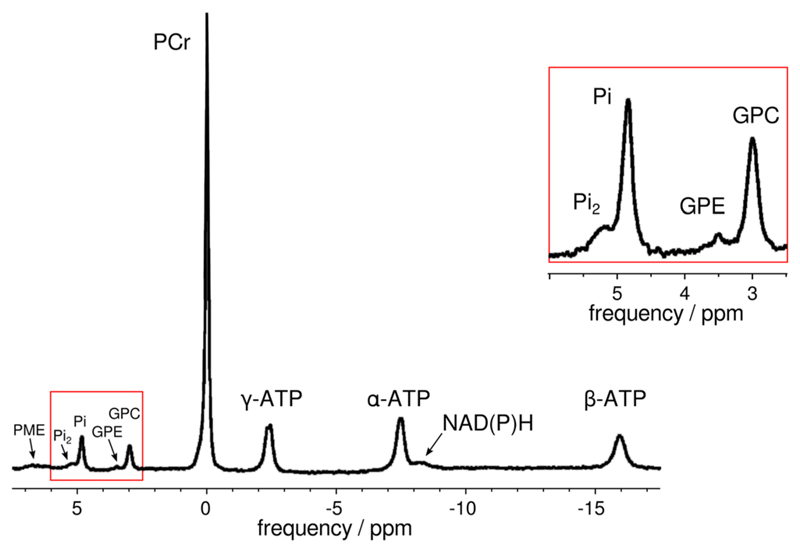
A typical ^31^P MR spectrum of the resting soleus muscle of a healthy volunteer acquired at 7 T, with the region between 2.5 and 6 ppm enlarged (right). Signals of an extra Pi pool and phosphodiesters (PDE) and phosphomonoesters (PME) are visible. Peak assignments: two signals for inorganic phosphate (Pi and Pi_2_), glycero-3-phosphocholine (GPC), glycero-3-phosphoethanolamine (GPE), phosphocreatine (PCr), three signals for ATP and pyridine nucleotides (NADPH/NADH). Data were acquired using a pulse-acquire sequence with a block pulse of 200 μs with a 5-cm surface-coil (*TR* = 5 s, bandwidth = 5 kHz, 2048 data points; 128 averages). Figure adapted from^50^

On most clinical MR systems, which are generally designed with ^1^H MRI as the main or only application, a package has to be acquired that allows ^31^P MRS. Such extensions generally enable the MR system to acquire signals from several ‘x-nuclei’ (i.e. nuclei other than ^1^H), and comprise additional hardware (usually a broadband amplifier, cabling, SAR supervision, receive system, and RF coils) and modifications of the scanner software. ^31^P MRS data acquisition should be optimised so that metabolites and derived measurements of interest (Table 1, Figure 1) are unambiguously detectable and quantifiable with sufficient SNR, while also fulfilling the demands imposed by the specificity of localisation, time resolution and exercise regime.

There are several aspects to consider:

The **radio frequency (RF) excitation pulse bandwidth** must be sufficiently large and the frequency profile should homogeneously excite all relevant metabolites for correct quantification. This is crucial for β-ATP, –16.26 ppm from PCr, if this resonance is to be used as a reference for absolute quantification^29^ (see also Table 2). Insufficient pulse bandwidth can produce strong chemical shift displacement artefacts when applying excitation with localisation gradients.

**Flip angles** of RF pulses should be known, as should the region over which the nominal flip angle applies when *B*_1_^+^ fields are inhomogeneous.

**Repetition time**: Signal averaging with partially-saturated spectra increases SNR per unit time, with Ernst angle excitation being preferable.^30^ While maximum SNR per unit time is achieved with shortest *TR* (and correspondingly the smallest Ernst angle),^31^ longer repetition times, on the order of metabolite *T*_1_ or more, are often chosen. This is advantageous because under partial saturation different *T*_1_ values of resonances (see Table 2) affect relative peak amplitudes, which requires correction for quantification (see section 2.3.3). At *TR* = *T*_1_ the theoretical signal reduction due to partial saturation is ~37 % with 90° excitation flip angle and ~27 % with the Ernst angle.

**Spectral resolution** must be high enough to resolve the metabolites of interest, for example PME, PDE, components of Pi or the split ATP resonances, (if measuring ^31^P-^31^P coupling constants or the phase evolution of the multiplets). This can also constrain the precision of pH quantification (see Table 1). If the chemical shift between Pi and PCr is measured in the spectral domain, zero-filling may enhance the nominal resolution in terms of Hz per spectral point in post-processing (section 2.3.1), and oversampling is often applied during acquisition but may be removed before data storage or data fitting.

**Echo time**: While *T*_2_ of most relevant metabolites is moderately long even at ultra-high field (> 100–400 ms, see Table 2), relatively short *T*_2_ relaxation times^32,33^ and homonuclear coupling of ATP leads to rapid signal decay after excitation,^34^ so non-echo-based MRS acquisitions with minimal acquisition delay are typically preferred for ^31^P MRS. Where echo-based acquisition is used, as in single voxel localisation in dynamic experiments,^34^ the echo time is preferably kept to a minimum and e.g. *TE* = 25 ms incurs only moderate signal loss for Pi at 7 T (*T*_2_ = 109 ms). ATP concentration was successfully quantified with *TE* = 7.4 ms at 3 T,^29^ while long *TE* requires long acquisition times (~20 min with *TE* = 110 ms for *T*_2_ measurements).^32^

**Shimming**: Narrow linewidth is of particular importance at lower field strengths, where the bandwidth is relatively low and metabolites can overlap, thus impacting their measured chemical shift (e.g. for Pi, which reduces the precision of the pH calculation). Whatever shim method is used, it is important for dynamic studies that the shim parameters are robust against motion, which can be facilitated by generous volumes to optimize field homogeneity.

**Nuclear Overhauser Effect** (NOE): SNR enhancement via heteronuclear ^1^H-^31^P NOE is achieved with RF pulses on the ^1^H channel during the parts of *TR* not used for ^31^P transmission and reception. To translate increased SNR into improved accuracy, the enhancement should be calibrated for the given setup in test measurements to evaluate efficiency and reproducibility for each metabolite. Magnetization transfer effects observed between ATP phosphates have been attributed to homonuclear ^31^P-^31^P NOE as a result of dipolar cross-relaxation within the phosphate spin system of ATP, due to its transient binding to slowly-tumbling large molecules.^35^

**^1^H decoupling**: Phosphate spins in mono- and diester groups are *J*-coupled with protons, which causes splitting of their resonances in the order of 7 Hz. As this splitting is not very well resolved it causes line broadening. By irradiation at the proper ^1^H frequency during acquisition it is possible to eliminate this coupling, which is particularly useful at field strengths of 3 T or below, where linewidths are in the order of the *J*-coupling. By ^1^H decoupling the signals of phosphocholine, phosphoethanolamine, GPC, GPE, α-ATP and NAD^+^ become much better detectable.^36^ ^1^H decoupling requires hardware adaptations to avoid ^1^H irradiation spoiling reception of the ^31^P signals.

**Localisation** can be implicitly set by the RF coil or explicitly defined via pulse sequences. Muscle ^31^P MRS is commonly, but not exclusively,^37,38^ performed with surface RF coils, which provide inherent localisation via the spatial profile of their RF (Tx and Rx) fields. Coil placement merits attention for several reasons. Firstly, during limb exercise, activation is muscle-specific,^34^ depends on the exercise paradigm,^39^ and is heterogeneous along the length of the muscle.^40^ Secondly, in resting muscle, it is important to know which muscle the signal originates from, as muscles may be affected differently in disease^41,42^ and may have different fibre-type compositions.^43^ Thirdly, because partial saturation depends on flip angle (which may vary over the sensitive volume), metabolite-specific *T*_1_, and *TR*, partial saturation may complicate (even relative) quantification of spectra; this can be remedied by localised acquisition schemes. Finally, when classical RF pulses are transmitted with surface coils, signal from superficial tissue may be partially suppressed when adjusting optimal excitation to deeper regions. Similarly, when employing adiabatic pulses to enlarge the effective region of optimum excitation to deeper regions, superficial regions are also excited at the nominal flip angle, which may be undesirable. When large coils that encompass several muscle groups are used, at least simple localisation should be applied^44,45^ to distinguish e.g. flexors from their antagonists (gastrocnemius and soleus vs. tibialis anterior in lower leg or the quadriceps and hamstrings in thigh) and muscles within a group that differ in fibre composition and contribute differently to exercise (like gastrocnemius and soleus in the calf).^39^ Several single-voxel^34,45^ and multi-voxel localisation approaches^39,42,46,47^ are available, each with specific advantages and drawbacks related to localisation power, time resolution, SNR, and ease of implementation. However, this is not required if the heterogeneity of the contributing tissue does not influence the interpretation of data and maximum SNR is critical,^48^ e.g. for PDE detection in small residual muscles of dystrophic patients.^49^ Optimal choice hence depends on the scientific question: see the following paragraphs on static and time-resolved dynamic MRS, and the scheme in section 2.4, Figure 4, for sensible combinations of techniques. In any case, realistic estimates of sensitive volume, contamination, and/or point spread function are necessary when designing a study.

#### 2.2.2 Studies in the resting state

At rest, longer acquisition times result in higher SNR, which allows detection of species with low abundance and visibility such as PME, PDE, a recently identified alkaline Pi_2_ peak,^49,50^ NAD(P)^+^/NAD(P)H and, indirectly, Mg^2+^ ^48^. It also allows higher-precision quantification of ATP, as a reference standard for absolute quantification in the analysis of a subsequent exercise bout. Resting state measurements can use localisation methods like ISIS or classical spectroscopic imaging (MRSI), which are available on most clinical MR scanners but require relatively long acquisition times, and are hence unsuitable for dynamic experiments. Care should be taken to choose sufficiently large matrix sizes (minimum recommended: 8 x 8) and appropriate Hamming weighting^51^ to minimise contamination, and the field-of-view should be large enough to avoid aliasing, *viz.* approx. 20 × 20 cm for the leg.

#### 2.2.3 Studies using magnetisation transfer

**Figure 2:**
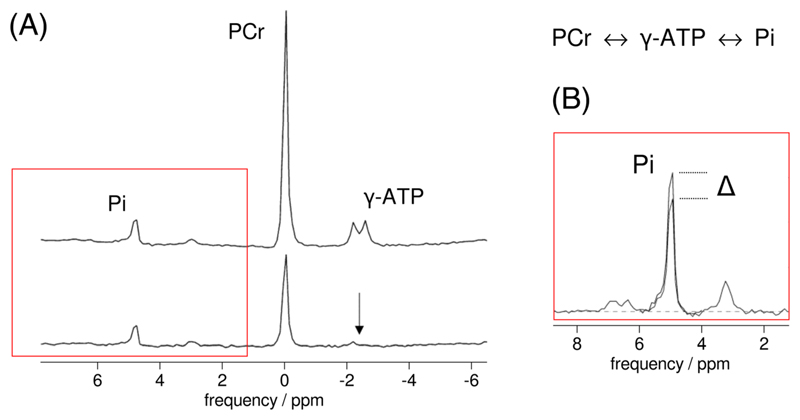
Spectra showing the principles of the saturation transfer experiment. In this example saturation of the γ-ATP resonance (A, lower) yields a reduction in the signals of Pi (and PCr) due to chemical spin exchange during the indicated reaction, as shown in detail for Pi in the insert (B), when compared to control conditions (A, upper); the difference Δ is then used to quantify Pi → ATP flux (see text). Figure adapted from^54^ which is licensed under CC-BY 3.0

Magnetisation transfer (MT) experiments concern the selective perturbation of the equilibrium magnetisation of one or more spin systems of metabolite nuclei and detecting the transfer of this perturbation by chemical spin exchange to the same nuclei in other metabolites. Transfer can also occur by cross-relaxation to nuclei at other positions in the same metabolite (i.e. homonuclear Overhauser effect). Selective perturbation can be performed by either spin saturation (saturation transfer, ST) or inversion (inversion transfer, IT), after which the transfer is monitored on the resonances of the exchanging nuclei. In ^31^P MRS, saturation transfer has been most widely employed,^52^ typically to measure Pi ↔ ATP and PCr — ATP exchange fluxes by saturating the γ-ATP spin pool and detecting differences in the signal of either PCr or Pi (Figure 2).

To quantify the Pi ↔ ATP exchange, the pseudo first-order rate constant (*k*’), which can be derived from the Bloch equations incorporating chemical exchange, can be calculated as *k*’ = (*M*_0_ – *M*_Z_) / (*M*_0_ *T*_1_*). In the case of measuring the Pi → ATP flux, *M*_Z_ and *M*_0_ are the equilibrium magnetisation of Pi under conditions of γ-ATP saturation and control respectively, and *T*_1_* is the apparent *T*_1_ of Pi in the presence of γ-ATP saturation, which generally has to be measured *in vivo* in an additional experiment. The Pi → ATP flux is then estimated by multiplying *k*’ by the concentration of Pi. Analogously, substituting for PCr signals and *T*_1_* in the equations yields an estimate of the PCr → ATP flux. For implementation, the selective saturation of γ-ATP is best achieved using a long, low-power, frequency selective pulse; however, when MR hardware precludes a long (many seconds) continuous pulse, as can be the case with clinical scanners, a train of shorter pulses with minimal inter-pulse delay is effective if the saturation profile is carefully optimised.^52,53^ Signal saturation is verified by checking nulling of the saturated resonance in spectra acquired *in vivo* (see Figure 2). Off-resonance effects of the saturation pulse have to be taken into account,^52^ e.g. by alternating this pulse between being centred on the γ-ATP resonance and at a frequency equidistant to Pi (or PCr), i.e. ‘mirrored’ around the resonance of interest.

As spectra are typically acquired using surface coils, *B*_1_ insensitive excitation and saturation pulses are preferred,^52^ and *TR* should be long enough to prevent artefacts arising due to differences in metabolite *T*_1_ values between conditions of control and saturation of γ-ATP. Many averages are generally required to accurately determine signal changes. Measurements in human skeletal muscle have typically been made during resting conditions, although the Pi → ATP flux has also been determined during steady-state exercise.^54,55^

In the interpretation of ST results the potential involvement of small pools of metabolites, competing exchange reactions and homonuclear NOE may have to be considered.^56,57^ For instance, effects on the signal of β-ATP after saturating γ-ATP were not due to chemical exchange, but were found to be an intramolecular ^31^P-^31^P NOE, which was assigned to the transient binding of ATP to large molecular structures in muscle cells.^35^ Furthermore, Pi ↔ ATP exchange may have multiple origins in the cell.^58^ To tackle the potential problem of analysing multiple (competing) reactions the saturation of multiple resonances in ST and wide band inversion in IT have been implemented.^52,59,60^

Although the potential of ST to detect exchange of small metabolite pools is of interest, it may be desirable to be sure that only MT effects among large pools are detected, which is achieved with IT methods. IT experiments have some advantages compared to ST experiments (e.g. no long saturation pulses, simultaneously measurable forward and reverse reactions), but the technique poses other challenges (e.g. *T*_2_ relaxation during the inversion pulse). The application of ST at 3 T turned out to be more robust than the applied IT method.^53^ Both ST and IT techniques are further developed to make them more efficient.^52,61^

#### 2.2.4 Dynamic (i.e. exercise/recovery) studies

Metabolic changes in muscle that can be observed with dynamic ^31^P MRS either occur on the time scale of a few seconds, such as pH at the onset and after cessation of exercise, or they have time constants of the order of half a minute, e.g. depletion of PCr during exercise and its post-exercise recovery, which can often be modelled as a mono-exponential function, or may have even longer time-courses e.g. post-exercise pH recovery. Hence, to capture changing pH and to reliably fit the PCr evolution with sufficient data points throughout exercise and recovery, the time resolution of repeatedly-acquired ^31^P spectra should be ~10 s or better. This temporal resolution necessitates shortening *TR* to the order of metabolite *T*_1_ values and accepting partial saturation.

**Figure 3:**
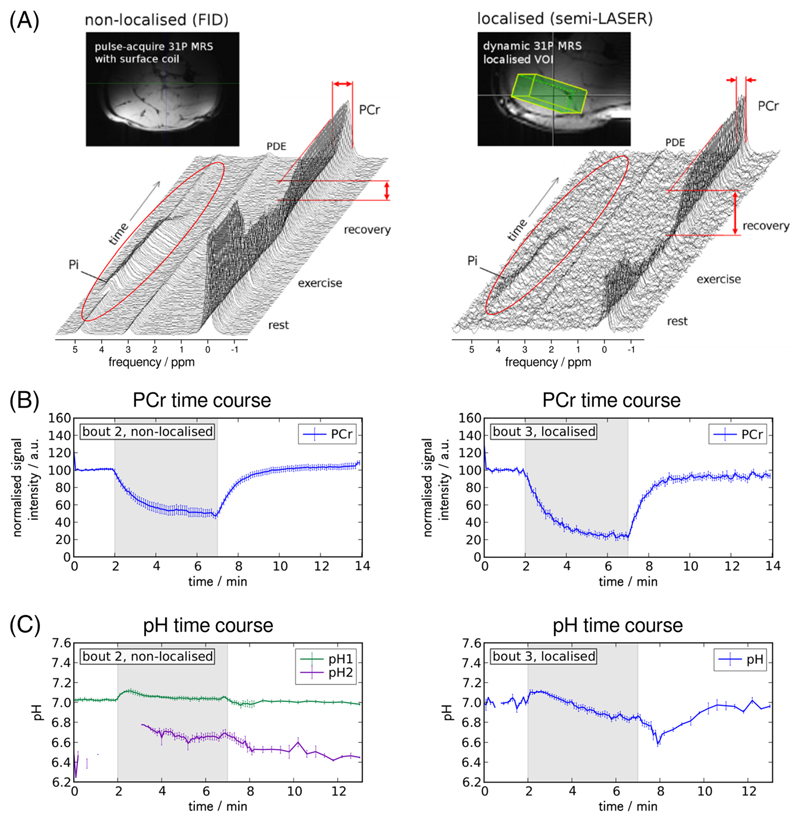
Time series of pulse-acquire spectra (A) measured at 7 T during rest, plantar flexion exercise and post-exercise recovery with a 10-cm surface coil placed below the calf and using a pulse-acquire scheme (250 μs block pulse) without further localisation (left) compared to semi-LASER single voxel localised MRS (*TE* = 23 ms) from the gastrocnemius medialis muscle (right). Both series: *TR* = 6 s, bandwidth = 5 kHz, 2048 data points; no averaging, 30 Hz apodisation. Non-localised spectra show higher SNR with broader linewidths but reflect less PCr depletion, as indicated by the arrows and visible in the time series of fitted PCr signal amplitudes (B). The inorganic phosphate peak is clearly detectable in all non-localised spectra, even at rest and during recovery, but is contaminated by signals from inactive tissue with neutral pH or shows a split peak (A), leading to ambiguous pH quantification during exercise and recovery (C). Figure adapted from,^44^ which is licensed under CC-BY-NC 2.5

Choice of voxel size or coil should minimise signal contamination from adjacent non-exercising muscle tissue, taking account of the point spread function and expected SNR (and hence feasible time resolution). Temporal SNR, the ratio of the mean signal amplitude over time to its standard deviation, is more important in dynamic studies than the SNR of each individual acquisition. A smaller sensitive volume generally gives narrower lines, improving SNR and unique identification of peaks; inclusion of inactive muscle tissue will impair quantification of exercise-related changes in PCr breakdown and pH (which may also become ambiguous due to Pi splitting, as demonstrated in Figure 3). Strictly, such partial volume effects should not affect measured *τ*_PCr_ (this being independent of absolute concentrations).^‡^

Practical aspects of exercising muscle in the scanner are considered later.

### 2.3 Data processing

#### 2.3.1 Preprocessing

When pulse-acquire techniques are used, the acquisition window may start too late to capture the first time points of the FID, especially when phase encoding gradients are following the excitation pulse or at higher field strengths where limited *B*_1_^+^ results in longer excitation pulses. This should be accounted for in post-processing, by adjusting the first order phase (or ‘begin time’ in time domain) before fitting. The nominal resolution of frequency spectra can be increased via ‘zero-filling’, i.e. appending nulls to the acquisition vector, although anything beyond doubling the vector size brings no real benefit, merely improving spectral appearance. Spectral SNR can be enhanced and baseline oscillations (from truncated FIDs) can be reduced by apodisation, at the cost of increased linewidth. Optimal SNR improvement is achieved with a ‘matched filter’, i.e. one that corresponds to the natural linewidth.

#### 2.3.2 Spectral fitting

Numerous tools are available for fitting ^31^P MRS data in time and frequency domains; however, few are well-suited to application to the large time-series of dynamic datasets. Popular software packages include jMRUI, OXSA, LC Model, TARQUIN and ACD Spectrus Platform.^62–65^ Important considerations when selecting a spectral fitting method for ^31^P MRS are its capacity for batch processing, ability to handle baseline problems, output format of results, and reported error estimates. The AMARES fitting algorithm provided in the jMRUI and OXSA platforms is readily applied in batch mode.^66^ Error estimates, particularly the Cramér–Rao lower bound, permit additional quality control of metabolite fits, though these should be interpreted with care.^67^

#### 2.3.3 Quantifying concentrations

In ^31^P MRS, there are several means of quantifying concentrations (cf. Table 4 and the footnotes therein) of phosphorus metabolites, including absolute quantification using internal and external references, and relative methods using metabolite ratios. In **relative methods**, metabolite concentrations are commonly represented by ratios to ATP or (less usefully, because this changes during exercise) PCr, or to total phosphate (the sum of all quantifiable phosphorus resonances in the ^31^P spectrum, which remains near-constant during typical exercise). ATP is most frequently used as an ‘internal’ concentration reference standard, as [ATP] is relatively consistent between individuals and differs relatively little between fibre types in humans; a normal resting ATP concentration of 8.2 mM is conventionally assumed.^29^ In the quantification of time-series data, normalising concentration to a low-SNR metabolite such as ATP can introduce more error than it is worth: it is better to assume constant [ATP] and either reference to ATP signal acquired with high SNR at rest, or to assume approximately constant total ^31^P signal.^§^ Most **internal-reference** methods have used ^1^H-MRS-measured tissue water as a reference standard, after correcting for sensitivity differences between ^31^P and ^1^H channels.^‖^ **External-reference** methods have used standards like phenylphosphonic acid, monopotassium phosphate or hexamethylphosphorous triamide (tris (dimethylamino)phosphine).^68^ These have been applied either in the same experiment, or in separate experiments with the same volume of interest; this necessitates matching coil-loading between muscle and a phantom, an external reference to account for load differences, or use of a *B*_1_ field map. An approach to account for varying coil-loading and receiver gains is to insert a synthetic reference signal via radiation (‘electronic reference to access in vivo concentrations’, ERETIC^69^) or inductive coupling.^70^ Taking full account of the many confounding factors makes absolute quantitation technically demanding.^71^ Because *T*_1_ and *T*_2_ differ between metabolites (see Table 2), all quantification strategies require **correction for saturation effects** (unless acquired under fully relaxed conditions) and for *T*_2_ (and *J* modulation of ATP) with echo-based acquisitions. Saturation correction can be done by taking the flip-angle dependent steady-state longitudinal magnetisation into account, using *M_z_*(*α*, *TR*) ∝ (1 - e^-*TR*/*T*1^) / (1 – cos *α* · e^-*TR*/*T*1^). While the correction for exponential *T*_2_-decay is straightforward (∝ e^-*TE*/*T*2^), the signal evolution with *J* depends on the pulse sequence and can be more complex than the cosine modulation applicable for a spin-echo sequence.

#### 2.3.4 Fitting time-series

Several approaches to quantifying mitochondrial oxidative capacity depend on fitting the PCr resonance during recovery from exercise, and thus, on determining the time or rate constant of PCr resynthesis. Robust fitting necessitates precise determination of the end of exercise, and assignment of spectra to the correct time points in case of time-averaged data. Including differently active muscle groups inside the field-of-view may lead to mixed, multicomponent recovery curves. Acidosis has a complex retarding effect on PCr recovery, leading to a multi-exponential presentation if signals from regions of tissue exercised at different extent are mixed. We recommend evaluating pH for all time points in the exercise interval; if the measured pH deviates by an amount greater than about 0.1–0.2 units from baseline (in practice this is impossible to define more closely), results should be interpreted with caution. In well-localised data, a mono-exponential fit is recommended (see Table 1), although in the presence of significant pH changes this no longer represents the underlying data well. Some investigators have proposed the use of bi-exponential or Weibull functions in these instances^72,73^ to extract the ‘early-recovery’ component, but these methods are not definitive.

### 2.4 Recommended combinations of instrumentation and RF pulse sequences

**Figure 4:**
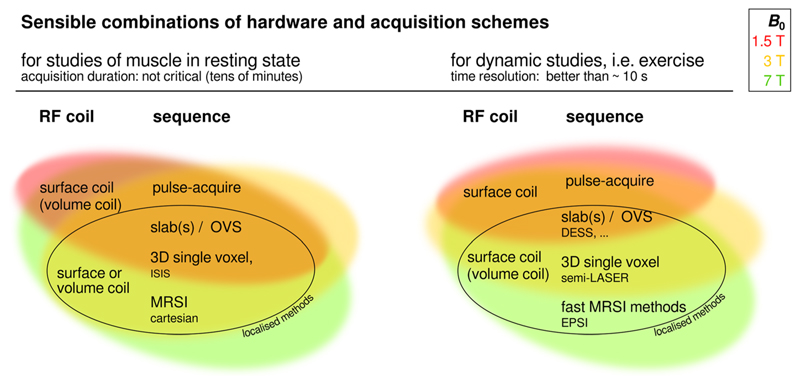
The figure shows combinations of RF coil and pulse sequence which are likely to be useful at different scanner field strengths (indicated by colour: see key). Requirements, and therefore recommendations, are different for static (left) and dynamic acquisitions (right). ‘Surface coil’ designates loop coils and coil arrays that provide some degree of localisation via their sensitive volume, while ‘volume coil’ designates birdcage coils and similar designs that can encompass e.g. a limb comprising several muscles or muscle groups. Parentheses indicate possible, but less favourable, combinations. The diagram should be read as follows: Dynamic studies employing localisation schemes are possible with sufficient SNR at high and ultra-high fields, preferably employing surface coils or arrays; at lower fields, employing a pulse-acquire scheme providing high SNR is preferable, relying on a surface coil for localisation. For studies of resting muscle, differentiation of individual muscles may be less critical, allowing for large volumes to contribute to the signal with large surface or volume coils, for high SNR, even at low fields

The technical requirements on ^31^P MRS data follow from the research question or application. Given that, different combinations of MRS methodologies can be recommended, within the constraints imposed by the available instrumentation (field strength, available RF coils) and, to a lesser extent, pulse sequences. Figure 4 gives an overview of recommended combinations for studies of resting muscle and for dynamic studies. Different quality in terms of SNR and hence feasible time resolution is to be expected from the different setups. The RF coil and its sensitive volume, voxel size and position, i.e. relative distance to the coil, have a strong influence on SNR with localising sequences, and some pulse sequences like classical MRSI with Cartesian read-out or 3D ISIS may not provide the required time resolution for dynamic acquisitions using standard exercise protocols, although a gated ^31^P 2D MRSI protocol has been implemented with repeated rapid dynamic contractions.^46,74^ Further influences are *TR, TE*, readout bandwidth and post-processing steps like the algorithm for combination of signals from different coil channels. Generally, the larger the signal-contributing volume, the larger is the SNR but besides the introduction of partial volume effects, linewidth increases. In Figure 4 coil types are separated into surface and volume coils, while array coils can fall into either of these categories. An array coil can provide the high SNR of surface coils or better, with a big field of view and homogeneous excitation via (static) *B*_1_^+^ shimming, depending on the coil design.

### 2.5 Typical values of measurements

As a practical guide to help in assessing implementation of experimental protocols, Table 2 gives typical values of some measured and calculated quantities in human skeletal muscle.

### 2.6 Reporting in publications

When reporting results it is important to consider what information is required for others to understand and follow to replicate the acquisition and quantification protocol. Not all parameters or equations need to be reported in the main text of every manuscript; referencing or inclusion as supplementary material is recommended.

Table 3 summarises the essential information that we recommend should be reported, and Table 4 gives the units in which the quantified metabolic parameters should be reported in publications, to allow straightforward comparison with the published literature.

## 3 MR and Non-MR Techniques Complementary to ^31^P MRS

Several techniques can help ^31^P MRS demarcate physiology from pathophysiology by providing information about blood flow and oxygen delivery/utilisation. Near-infrared spectroscopy (NIRS) can assess relative concentration changes in oxygenated, deoxygenated and total haem. Unfortunately, the NIRS signals from (intracellular) myoglobin (Mb) and (intravascular) haemoglobin (Hb) overlap. Conventional analysis attributed the muscle signal to Hb.^75^ Recent work combining NIRS with ^1^H MRS, which can distinguish Mb and Hb signals, has now clarified these contributions: NIRS mainly reports the oxygenation of Mb.^76–78^ Combining NIRS and ^31^P MRS offers an opportunity to better understand adaptation and capacity in contracting muscle.^79^

The use of simultaneous measures of electromyography and ^31^P MRS can be used to identify the mechanisms of muscle fatigue *in vivo* and improve interpretation of the metabolic responses to incomplete voluntary activation of skeletal muscle.^80^

Arterial spin labelling (ASL) MRI assesses blood perfusion^81^ and blood oxygen level dependent (BOLD) imaging can monitor regional oxygen changes.^82^ Interpreting BOLD requires caution, because many confounding factors can affect the *T*_2_* weighted images,^83^ notably pH change.^84^ To reduce potential confounding variables, protocols consisting of brief contractions have been developed.^85^

Acquiring simultaneously or interleaved ^1^H MR and ^31^P MR signals enables the capture of complementary metabolic information during a single exercise bout.^83^ Studies have combined ^31^P MRS with ^1^H MR to measure BOLD signals,^82,86^ perfusion,^87–89^ Mb and intracellular O_2_,^76,87,90^ lactate^87,91^ and most recently carnosine.^92^ Such interleaved measurements require modification of pulse programs and sometimes hardware.^89,93^

Finally, metabolite-specific ^31^P MRI can localise metabolite signals and pH within a tissue region,^47,94^ and new ideas such as fingerprinting and artificial intelligence-based approaches for ^31^P and metabolite kinetics are being developed, but this topic extends beyond the present scope.

## 4 Important Non-MR Factors in Dynamic Muscle ^31^P MR Studies

### 4.1 Muscle, muscle size, mode of exercise

The choice of muscle will determine the choice of exercise and vice versa. Different ways to apply exercise load range from simple rubber bands, through lifting of weights, to highly sophisticated ergometers.^95,96^ A factor to consider in the interpretation is the size of the recruited muscle affecting the observed metabolic signals ([CO_2_], [H^+^], lactate, [O_2_], free radicals) involved in the homeostatic cardiovascular and ventilatory responses.^97^ Another is the degree of eccentric vs. isometric/concentric exercise, as their molecular mechanisms differ,^98^ which results in different haemodynamic and metabolic responses.^99^

Determining contraction intensity is a pre-requisite for in-magnet exercise studies, especially those that relate intensity to changes in PCr or similar measurements. On-line monitoring of the subject's activity and storage of these motion data is desirable, as it allows monitoring the subject's compliance to the protocol, ensures correct assignment of exercise and recovery phases, and identifies motion artefacts, all of which helps to improve data quality. However, accurate load measurement in the MR environment via sensors capturing force and motion is not trivial, and requires dedicated MR-compatible systems (e.g. optical equipment). The heterogeneity of muscle recruitment needs to be considered in the interpretation of exercise-induced metabolic changes, as it can be highly inhomogeneous, e.g. even among plantar flexors^39^ and along muscles,^40,89^ as recent localised 7 T experiments have shown. The scope for extraneous movements must be minimised. Comfortable yet tight fixation and careful reproduction of the positioning between subjects in longitudinal studies will contribute to reliability. Exact adherence to exercise timing is crucial (e.g. a ‘clean’ cessation for measurement of PCr recovery kinetics). Better protocol adherence can be obtained with electrostimulation; however, temporal and spatial recruitment differ substantially from voluntary contractions and result in different haemodynamics and metabolic perturbations. While motor nerve stimulation can activate all motor units, it can be problematic (activating antagonists, being painful or increasing risk of injury). In contrast, motor point stimulation activates only a portion of the muscle.

### 4.2 PCr recovery kinetics

Mono-exponential PCr recovery^12^ is less dependent on exact exercise intensity than methods that study the PCr decrease or Pi increase as a function of load. To measure PCr recovery kinetics, the exercise bouts must be intense enough to induce a substantial (30–40 %) PCr depletion while pH should not decrease more than 0.1 – ~0.2 units, as this complicates the kinetics and interpretation of PCr recovery (see above).^14^ To achieve this, a preliminary incremental/ramp protocol can be used to determine the workload corresponding to the onset of acidosis^100^; alternatively, each subject's maximum voluntary force may be determined to scale the workload, though this may not be feasible in some patient populations. Use of relatively brief, maximal voluntary contractions ensures that all motor units are activated while keeping acidosis to a minimum.^101^ A different approach to measuring PCr recovery kinetics without complicating pH change is to use brief ‘pulses’ of muscle stimulation, multiply-averaged to improve SNR (usefully, this also allows estimation of ATP usage rate during the stimulation (exercise) period).^46^ Reproducibility of PCr recovery kinetics can be optimised with some warm-up exercise.^102^

It is important that the experimental setup is not allowed to influence muscle blood flow (e.g. hindering it by fixed joint position or isometric/eccentric load). In the extreme case, stoppage of blood flow by cuff ischaemia will completely stop PCr recovery.^103^

### 4.3 Recommended steps of a dynamic MR examination

For a dynamic MR examination we recommend evaluating the clinical status of the subjects and their ability to undergo the exercise. Next consider the choice of parameters that can be measured using an available ergometer. Finally, adjust the dynamic protocol (i.e. with both concentric and eccentric phases) to suit the subjects and the available ergometer.

It is desirable that a test–retest should be performed and reported for each specific protocol.^95,104^

Reliable examinations depend critically on a reproducible setup, standardised preconditioning of the subject, and control of potential difficulties. Table 5 lists some relevant considerations and potential confounders; these may be unavoidable, but should be documented in ‘Material and Methods’ or the ‘Discussion’ section.

## 5 Data Interpretation

### 5.1 Interpreting resting data

In general the resting values of quantities measured by ^31^P MRS are set by an interacting combination of mechanisms including the kinetic properties of transmembrane transport of Pi, creatine, and H^+^, and the regulation of basal ATP synthesis rate.^14,105,106^ Any of these might differ between fibre types, with training state or age, and in disease.

Resting metabolite concentrations differ between myofibre subtypes (more so in rodents than human),^29^ and so inferences about fibre-type composition have been made on the basis of resting PCr/Pi and PCr/ATP ratios, albeit with differing findings.^107,108^

The lower PCr/ATP and PCr/Pi ratios and higher Pi/ATP seen in resting muscles of patients with genetic defects in mitochondrial oxidative ADP phosphorylation^109^ can largely be explained in terms of the primary pathology.^14^ In muscular dystrophies elevated resting intramuscular pH ^110,111^ probably relates to membrane leakage and sodium accumulation with associated ‘compensatory’ proton extrusion; in some patients, multiple Pi resonances suggest pH heterogeneity.^49^ Increased PDE/ATP ratios in muscular dystrophy,^38,111^ fibromyalgia^109,112^ and the elderly^113^ are thought to reflect elevated membrane turnover and disturbed phospholipid metabolism.^114^ Free intramuscular Mg^2+^ concentration is decreased in Duchenne muscular dystrophy,^48^ a likely consequence of membrane leakiness.

### 5.2 Interpreting PCr kinetics during exercise and recovery: Mitochondrial function

The simplest cases of exercise protocols are ‘pure oxidative’ exercise at constant power, or recovery from such exercise, where the rate constant of the change in PCr (decrease during exercise, resynthesis during recovery) is proportional to the mitochondrial capacity measured in various other ways.^115–117^ This interpretation is complicated when there is pronounced pH change during exercise due to significant non-oxidative glycolytic contribution to ATP synthesis. Kinetics of PCr change during exercise then become an unreliable quantitative guide to mitochondrial function (although impaired mitochondrial function is likely to lead, other things being equal, to greater changes in PCr during exercise). Furthermore, in recovery from exercise with a physiologically significant pH decrease (say >0.2), the interactions between pH, ADP and PCr concentrations via the CK equilibrium result in a relationship between end-exercise pH and PCr recovery kinetics (lower pH, slower recovery), independent of changes in mitochondrial capacity.^118–120^ Various ways, with some theoretical support and proven empirical utility, have been devised to correct for this effect.^14^ Some of these methods of calculation and interpretation yield estimates of mitochondrial capacity in units of absolute metabolic flux, but their relationship to measures made by invasive physiological or *ex vivo* biochemical measurements is not yet completely understood.^14^

Conducting the exercise so as to minimise muscle acidification allows simply using the rate constant of PCr recovery as a measure of whole-muscle oxidative capacity, rather than ‘mitochondrial capacity’, *per se.* ^121^ This is a system property with contributions from a number of factors including the number of mitochondria, the amount and the activity per mitochondrion of respiratory chain components and enzymes of fat and carbohydrate oxidation, but also the vascular supply of O_2_, and the diffusion of O_2_ across the capillary wall and through the myocyte to the mitochondria. A slow PCr recovery may reflect impairment of any of these processes.^14^ Situations in which O_2_ availability is changed, such as in peripheral vascular disease,^122^ reactive hyperaemia,^123^ experimental hypoxia in untrained subjects,^124^ and chronic obstructive pulmonary disease,^125^ are particularly likely to be confounded. However, in the submaximal exercise typically used in ^31^P MRS work, one would not (in normoxia) expect whole-body cardiovascular or respiratory function to affect ^31^P MRS measures of mitochondrial function, and the relevant factors are distal to the artery supplying the muscle studied.^14^

### 5.3 Interpreting other features of dynamic ^31^P MRS studies

The assessment of contractile cost from the initial rate of PCr depletion using exercise is reasonably uncontroversial, providing a reliable measure of mechanical output is available. This is an interesting and potentially useful physiological property,^46^ but relatively under-studied.

Changes in pH during exercise and recovery depend on passive buffering processes, the acidifying effect of glycolytic ATP synthesis (an accompaniment of lactate production) and the pH-restoring effects of processes of acid efflux. Although the principles are reasonably clear,^106^ the quantitative details are not necessarily well understood, and physiological validation by other methods is rare. In some cases the (patho)physiological interpretation is straightforward. For example, if glycogenolysis is absent, as in the metabolic disorder McArdle's disease (muscle glycogen phosphorylase deficiency), exercise produces a characteristic and quantifiable pattern of ^31^P MRS abnormalities.^126^ If more subtle changes in glycogenolysis are of interest, it makes sense to study the muscle in ischaemic exercise, where there is no oxidative contribution to ATP synthesis.^26^ Another simple example: when peripheral vascular disease impairs the ability to clear acid from the muscle cell, pH recovery after exercise is slowed,^122^ pH and PCr recovery kinetics can be used to estimate absolute rates of post-exercise acid efflux^14,21^ but this has rarely been exploited in disease.

In acidifying exercise the presence of different-pH components as ‘splitting’ of the cytosolic Pi resonance may be an index of different responses by the various myofibre types,^127,128^ provided localisation is adequate to ensure that the heterogeneity is within a single muscle.^44,129^ Inference must be very cautious here.

### 5.4 Interpreting magnetisation transfer measurements

Pi → ATP flux measured by MT in resting muscle has been suggested to reflect mainly oxidative ATP synthesis, on the two assumptions that this is unidirectional (so that exchange flux ≈ net rate of ATP synthesis) and that other contributions (e.g. near-equilibrium exchange via the glycolytic enzymes GAPDH and PGK) are relatively small.^130^ However, observed rates of Pi → ATP flux are much larger than known rates of oxidative ATP synthesis in resting muscle, so one or both assumptions must be wrong.^58^ Recent measurements of Pi → ATP flux during steady-state exercise in human muscle show that this discrepancy is approximately independent of ATP turnover.^54^ Despite these physiological uncertainties, which argue against any simple conceptual relationship between the two quantities, resting Pi → ATP flux was previously proposed to be an indirect measure of mitochondrial capacity. It is unsurprising that some studies show no empirical relationship between them. More puzzlingly, some studies do show some interesting correlations between resting Pi → ATP flux and measures of resting ATP turnover and mitochondrial capacity^131^; the physiological basis of these remains unexplained.^54^

## 6 Conclusions

Skeletal muscle ^31^P MR spectroscopy can provide insights, not otherwise available non-invasively, into the regulation and pathophysiology of what may be summarised as cellular energy metabolism or ‘bioenergetics’: the production and use of ATP. Most common is the use of voluntary exercise or electrical stimulation as a dynamic probe to assess the metabolic response to increased workload. The post-exercise kinetics of PCr resynthesis offer the most straightforward way of quantifying the rate and capacity of mitochondrial ATP synthesis, best considered as a system function of the organ and its blood supply. Changes in cytosolic pH reflect the balance of anaerobic glycolytic ATP synthesis and the processes of acid efflux. The use of ^31^P MRS in resting muscle can profit from increased SNR due to longer acquisition times, which allows relatively easy application of localisation schemes. This has been exploited particularly for studying various diseases. Combining ^31^P MRS with other methods can add valuable complementary information on O_2_ delivery, amongst other things.

The recommendations given here, of which the most important ones are listed in Table 6, are intended to guide those who have experience in general MRS to the special application of ^31^P MRS in skeletal muscle, covering the practicalities of acquisition and exercise as well as the physiological interpretation of the measurements.

## Supplementary Material

Appendix A

## Figures and Tables

**Table 1:** Quantities assessable with ^31^P MRS, and some derived metabolic quantities, pitfalls in data acquisition and possible remedies. Values are given for resting state, except where indicated

Measured metabolite	Challenges and pitfalls		Remedy or mitigation
Phosphocreatine (PCr)	Long *T*_1_ relaxation time, but *decreasing T*_1_ at ultra-high field^33^		Scan at the Ernst angle

Adenosine triphosphate (ATP)	Concentration low (SNR) → may affect accuracy of all metabolites if used for absolute quantification		Quantify ATP from averaged resting data
	Decreased visibility due to *J*-coupling and *T*_2_ relaxation (particularly at ultra-high field) with echo-based methods		Use shortest possible *TE* (additional ATP quantification at rest with zero echo time sequence is possible, but almost never done)
	Chemical shift (for β-ATP) → decreased visibility due to excitation pulse bandwidth (hence also different *T*_1_ weighting) or chemical shift displacement artefact with some localising sequences		Use γ-ATP instead

Inorganic phosphate (Pi)	Concentration low (SNR)		Use appropriate averaging
	Decreased post-exercise visibility due to rapid concentration decrease, peak splitting or linewidth increase, either as consequence of partial volume effect (artefact) or as expected effect of exercise		Average for pH quantification with lower time resolution during recovery^44^ (see Figure 3c)
	Splitting/detection of acidotic Pi resonance during/after exercise: broadening due to partial volume artefact or true heterogeneity of fibre composition		Use appropriate localisation to avoid partial volume effect; identify true heterogeneity/compartmentation
	Splitting/detection of alkaline Pi resonance at rest (mitochondrial^50^ or extracellular/interstitial^49,132^: low concentration, separation from main Pi peak)		Use averaging, improve linewidth by shimming (*B*_0_-map, FASTMAP); scan at ultra-high field
	Long *T*_1_ relaxation time, which does *not* decrease at ultra-high field^33^		Scan at the Ernst angle

Phosphodiesters (PDE)	Concentration low (SNR)		Use appropriate averaging
	Specificity: PDE = combined signal of GPE and GPC		Use ^1^H decoupling; scan at ultra-high field; improve linewidth by shimming

PME	Concentration low (SNR), broad signal		Use appropriate averaging; use ^1^H decoupling

NAD^+^/NADH and NADP^+^/NADPH	Concentration low (SNR), impaired detectability. Appears as shoulder on α-ATP, hard to separate. Assignment of multiple peaks to metabolites and compartmentation.^133^		Use appropriate averaging; improve linewidth by shimming; use appropriate localisation; use ^1^H decoupling (decreases α-ATP and NAD^+^ linewidth

**Derived quantity**		**Challenges and pitfalls**	**Remedy or mitigation**

pH	Chemical shift *δ* between Pi and PCr using Henderson-Hasselbalch equation^134^: pH = 6.75 + log[(*δ* – 3.27)/(5.63 – *δ*)]	Broad or split Pi peak	For two peaks: pH of separate peaks,^50,132^ or weighted combination of both Pi peaks^132^ For one broad asymmetric peak: weight according to frequency ranges and amplitudes of Pi moieties
		Spectral frequency resolution	Use time-domain fitting; increase spectral resolution in acquisition In frequency domain: use up to 2× zero-filling with apodisation

Free [ADP]	Adenosine diphosphate concentration from pH and [PCr] assuming creatine kinase (CK) equilibrium^106^: [ADP] = {([TCr]/[PCr]) – 1} · [ATP]/(*K*[H^+^])	Assuming normal total creatine concentration ([TCr]) may be wrong, especially in disease or altered dietary creatine	Measure [TCr] in parallel or separate experiments by ^1^H MRS or biopsy^29^
	Where *K* = 1.66 × 10^9^ l mol^–1^ and normal [ATP] and [TCr] = 8.2 and 42.5 mmol/l cell water, respectively^29^	The expression is an approximationc	More complex expressions are available^26^
		The calculation of ADP assumes free solution in the cytosol; recent work^35^ calls this into question	

Δ*G*_ATP_	Gibbs free energy of ATP hydrolysis^106^:Δ*G*_ATP_ = Δ*G* ^0’^_ATP_ + *RT* ln([ATP][Pi]/[ATP]) Where Δ*G* ^0’^_ATP_ = 32 kJ mol^–1^ and *RT* (gas constant × temperature) = 2.57 kJ mol^–1^	Same limitations and mitigations as its component measurements (q.v.)	

Mg^2+^	Chemical shift *δ* between α-ATP and β-ATP^135^ or between PCr and β-ATP^136^	Confounders of α-ATP and β-ATP (broad or unresolved resonances)	Improve linewidth by shimming; use averaging; ensure sufficient spectral resolution
		pH-dependent, requires assumptions for exchange between Mg^2+^, H^+^ and ATP^136^	Determine pH robustly; assume standard values for the different exchange variables^48^

PCr recovery kinetics	PCr(*t*) = PCr_e_ – ΔPCr · exp(–*t*/*τ*_PCr_) where PCr_e_ is the [PCr] after recovery, ΔPCr is the difference between post-recovery and post-exercise [PCr], and *τ*_PCr_ is the time constant for PCr resynthesis. The rate constant is defined as *k*_PCr_ = 1/*τ*_PCr_, and the half-time as *t*_1/2_ = ln(2) *τ*_PCr_	SNR or time resolution	Use maximum reasonable voxel size; avoid partial volume effects; improve linewidth by shimming
		Signal instability of PCr or total ^31^P signal during the time-course, especially at end of exercise and after recovery	Minimise gross motion using straps and pads for subject positioning; give subject clear instructions
		Multi-exponentiality, partial volume effects, (partial) acidification^120^	Use localisation; keep exercise submaximal; use more complex fits
	Various approaches to the apparent maximum rate of oxidative ATP synthesis *Q*_max_^14,106^	Absolute values depend on theoretical framework and assumed parameters^14^	Use relative changes (less sensitive to these confounders)

H^+^ efflux rate	Calculated from pH and d[PCr]/d*t* in recovery from exercise^106^	Assumptions about buffer capacity *β*	Assume standard or indirectly-measured *β*,^26^ or determine *β* separately using lactate ^1^H/^31^P MRS^91^

**Table 2:** Typical ^31^P MRS skeletal muscle measurements. Metabolite quantities are reported as signal ratios and were acquired under fully relaxed conditions or corrected for partial saturation

Measure	Reported mean values in healthy cohorts *		Possible deviations in disease and other comments ^†^			

***Calf muscle***						

Resting muscle						

PCr/ATP	4.23 ± 0.24 (8) [3.22–5.20]		Large variation in both health and disease; can decrease by up to 50 % in some diseases			

Pi/ATP	0.56 ± 0.13 (8) [0.37–0.81]		[0.75–0.85] in various diseases			

PDE/ATP	0.12 ± 0.04 (5) [0.13–0.32] children 0.19 ± 0.05 (5) [0.07–0.43] adult		Increases with age; can increase in some diseases, as much as 2–3 times in dystrophic muscle			

Pi/PCr ^‡^	0.13 ± 0.01 (8) [0.09–0.17]		[0.18–0.20] in various diseases, e.g. high (~0.60) in dystrophic muscle			

pH	7.03 ± 0.01 (10) [7.01–7.08]		Increased (> 7.08) in some diseases e.g. up to 7.40 in dystrophic muscle			

Post-exercise PCr recovery kinetics						

*τ*_PCr_ (without acidification) ^§^	41 ± 3 s (5) [31–50 s]		Up to ~60 s in some diseases			

*Q*_max_	0.5–0.9 mM/s ^14^		Sensitive to model and assumptions underlying the calculation			

***Thigh muscle (quadriceps/hamstrings)***						

Resting muscle						

PCr/ATP	4.48 ± 0.20 (9) [3.81–5.80]		Large variation in health and disease			

Pi/ATP	0.48 ± 0.05 (5) [0.33–0.60]		[0.65–0.75] in various diseases			

PDE/ATP	0.32 ± 0.11 (4) [0.09–0.65] adult 0.49± 0.14 (2) [0.18–0.80] elderly		Increases with age (up to 50 % increase between young adults and elderly); can increase 25–40 % in some diseases			

Pi/PCr ^‡^	0.11 ± 0.01 (5) [0.09–0.13]		[0.15–0.18], increased in some diseases, e.g. ~0.5 in dystrophic muscle			

pH	7.05 ± 0.01 (8) [7.01–7.14]		In patient groups > 7.08; can reach 7.40 in e.g. dystrophic muscle			

Post-exercise PCr recovery kinetics						

*τ*_PCr_ (without acidification) ^§^	26 ± 1 s (6) [23–29 s]		Up to ~50 s in disease without significant acidification during exercise			

*Q*_max_	0.5–0.9 mM/s ^14^		Sensitive to model and assumptions			

***Relaxation times of most abundant metabolites***						

	**1.5 T** ^33^		**3 T** ^32,33^		**7 T** ^33,137^	

*Metabolite*	*T*_1_/s	*T*_2_/ms	*T*_1_/s	*T*_2_/ms	*T*_1_/s	*T*_2_/ms

PCr	5.7 ± 0.6 (5)	425 ± 1 (2)	6.6 ± 0.2 (2)	344 ± 14 (2)	4.0 ± 0.2 (2)	217 ± 14 (1)

γ-ATP	4.4 ± 0.3 (5)	93 ± 3 (1)	5.0 ± 0.7 (2)	70 ± 11 (2)	3.7 ± 0.6 (2)	29 ± 3 (1)

α-ATP	3.4 ± 0.4 (5)	74 ± 1 (1)	3.0 ± 0.5 (2)	51 ± 6 (2)	1.8 ± 0.1 (2)	-

β-ATP	3.9 ± 0.3 (5)	75 ± 2 (1)	3.7 ± 0.3 (2)	55 ± 10 (1)	1.6 ± 0.3 (2)	-

Pi	4.3 ± 0.6 (5)	223 ± 25 (2)	6.1 ± 1.2 (2)	151 ± 4 (2)	6.5 ± 1 ** (2)	109 ± 17 (1)

PDE	-	-	8.6 ± 1.2 (1)	414 ± 128 (1)	5.7 ± 1.5 (1)	314 ± 35 (1)

PME	-	-	8.1 ± 1.7 (1)	-	3.1 ± 0.9 (1)	-

* The values in this column are the mean ± SEM in (*n*) studies [range of means], given as an indication of consensus. In the majority of these studies, data were acquired under similar conditions (surface coils, no echo-time), and all were corrected for metabolite *T*_1_, if applicable.

† This column aims to give an approximate indication, where possible, of how abnormal the different measurements can be in various disease states, and in which direction; the actual abnormalities in any measurement will of course depend on the particular pathophysiology.

‡ When not reported this was calculated from the study mean Pi/ATP and PCr/ATP. Absolute concentrations often are calculated assuming constant [ATP] with the standard value of 8.2 mM, rather than being measured directly.

§ Halftime and rate constant of PCr recovery can be calculated from this as in Table 1.

** For the alkaline inorganic phosphate component Pi_2_ attributed to a mitochondrial origin shorter *T*_1_ of 1.4 ± 0.5 s was reported.^50^

**Table 3:** Minimum requirements for reporting acquisition and data processing parameters

General parameters	
Hardware	MR scanner: field strength, gradient strength and slew rate if appropriate. RF coil type, size and geometry RF coil transmit *B*_1_ and estimated sensitive volume (with technique used to measure/simulate *B*_1_^+^ and determine excitation flip angle) Any additional equipment e.g. ergometer, 2^nd^ RF (Tx/Rx) channel
VOI, positioning and shim	If a localisation sequence is used: the position and size of the VOI Otherwise: the position of the RF coil in relation to muscle anatomy The point spread function (which influences contamination from surrounding tissue, and thus the effective VOI size) Method of *B*_0_ shimming (including e.g. VOI size)
Acquisition sequence	Type of sequence Sequence timings, e.g. *TR*, *TE*, *TM* Number of averages, acquisition bandwidth, vector size (and resulting total acquisition duration) Shape, duration and effective flip angle of all relevant pulses along with (or allowing for calculation of) the bandwidth as well as potential chemical shift displacement artefact
Data exclusion criteria	e.g. SNR, linewidth or minimum change in metabolite concentration
Data quantification	Processing steps and parameters: zero-filling, truncation, apodisation function Type of fitting algorithm/software used, fitted line shape (e.g. Lorentzian or Gaussian) Prior knowledge used (if applicable) If absolute quantification of metabolite concentrations was performed, what was used as internal/external reference Correction for partial saturation (saturation correction factors)
**Additional parameters for dynamic examinations**	
Temporal resolution	Related to acquisition and whether data averaging was used
Exercise task and study protocol	Duration of exercise and recovery blocks Type and intensity of the exercise Additional information about calibration of workload e.g. what percentage of maximum voluntary contraction (MVC) force or power, also how MVC was determined Technique for exercise and acquisition synchronisation
Participant preparation	e.g. through detailed description, separate study day visit or a video
Data quantification	How was recovery fitted and what model was used to calculate *Q*_max_
**Additional parameters for saturation transfer (ST) examinations**	
ST at rest	Saturation pulse/train length and bandwidth Saturation frequency of the saturation and control experiment Method used for *T*_1_ measurement
ST during exercise	Timing of the acquisition: how soon after exercise onset was the ST acquisition; performed within one or split over several exercise bouts

**Table 4:** Recommended forms of the quantified metabolic measurements

Measurement	Units to be reported
Measured concentrations of Pi, PCr, ATP, Mg^2+^, PDE, (PME)	mM *
Calculated concentration of free ADP ^†^	μM
PCr recovery time constant *τ*_PCr_ or halftime *t*_1/2_	s
Exchange rate constants *k*, PCr recovery rate constant *k*_PCr_	s^−1^
Initial PCr recovery rate *V*_PCr_	mM/s
Mitochondrial oxidative capacity *Q*_max_	mM/s
Metabolic fluxes	mM/s

* Metabolite concentrations in mmol/l cytosolic water are sometimes written as mmol/l or simply mM. Also mmol/kg wet tissue is used in the literature, but this should be defined if used. We use mM in the sense mmol/l cytosolic water for the flux measurements later in the table. The relation between these units is described elsewhere.^29^ To what extent ^31^P MR-detectable metabolites are straightforwardly free in cytosolic aqueous solution is an empirical question,^138^ although for practical purposes is often simply assumed.

† As the calculation is based on a cytosolic equilibrium assumption, it is natural to use cytosolic water as the denominator.

**Table 5:** Necessary considerations for experimental design and potential confounders to be documented in publication

**Factors to consider in the experimental design**
Muscle size and metabolic characteristics
Concentric vs. eccentric workload = different energy demand
Isometric vs. isotonic workload = different energy demand (also prolonged isometric exercise may compromise vascular O_2_ supply.)
Exercise intensity and exercise timing – Maximum voluntary force
**Potential confounders**
Muscle(s) recruited during the movement or activated by the stimulated nerve (i.e. proportion of active versus inactive muscle contributing to spectra)
Extraneous movement (adapted positioning/fixation)
Changes in sensitive volume due to motion
Quantification of mechanical work missing or attribution to individual muscles uncertain
Load- and pH-dependent PCr recovery kinetics
Influence of O_2_ availability on recovery (vascular disease, eccentric workload)
Other biological confounders (e.g. health/disease, diet, medication, regular/exceptional physical activity, training status)

**Table 6:** Summary of main recommendations. This table is intended to guide scientists experienced in MRS to the specific application of ^31^P MRS in skeletal muscle. It deals with the most important, or least obvious, aspects of data acquisition and post-processing, and gives practical advice on equipment setup, preparation of subjects and performance of exercise. For details, further recommendations and aspects of physiological interpretation, see main text of the indicated sections

Problem/field	Recommendation	Refer to
Choice of sequence, parameters and instrumentation	The scientific question determines the metabolites of interest, minimum required SNR, volume of interest, and time resolution (in dynamic studies); tailor technique accordingly, considering parameter space and boundary conditions. Prioritise: optimise important measurements, avoid unnecessary ones (e.g. [ATP] when the focus is on kinetics).	Section 2, esp. 2.2, 2.4
SNR and temporal resolution	Use appropriate combination of coil, field strength, sequence and parameters, e.g. measurement volume, *TR*, flip angle.	Section 2.2, Figure 4
Use of NOE	Perform calibration measurements per metabolite *in vivo.*	Section 2.2
Partial volume effects	Localise by sufficiently small surface coil (correct placement, superficial muscles), single-voxel or MRSI. Make realistic estimates of sensitive volume. Consider which muscles are exercising or affected by disease.	Section 2.2
MRSI acquisition	Use minimum matrix size for acceptable resolution, spatial response function, partial volume effects, SNR/measurement time.	Section 2.2.2
Magnetisation transfer	Ensure adequate saturation, sufficient *TR*, high-quality *T*_1_ measurements. Account for off-resonance effects, competing exchange reactions and metabolite pools.	Section 2.2.3
Acquisition of PCr recovery data	Ensure sufficient PCr depletion (depending on time-series SNR) and time resolution (≤ 10 s). If using first-order model to quantify mitochondrial function (*τ*_PCr_, halftime or rate constant) keep exercise pH change small (≲ 0.2 units).	Section 4, esp. 4.1
Quantification of spectra	Quantify spectra as area of peak (fit in time- or frequency-domain or integrate peaks). Correct for saturation. Use ATP from high-SNR (resting) spectra as internal reference. Detect and fit split resonances (Pi) and multiplets (ATP) for accurate pH quantification and fit fidelity.	Sections 2.2.1, 2.3.3, Table 2
Quantifying recovery kinetics	Correctly define end-exercise time point and timing of averaged blocks. If exercise pH change ≳ 0.2 units, take account by appropriate model/calculation (e.g. *Q*_max_).	Section 2.3.4
Exercise design	Consider prescription and monitoring of exercise type, timing and force. Standardise preconditioning and feedback to subject during exercise.	Section 4, esp. 4.3, Table 5
Confounders for exercise protocols	Document confounders, e.g. heterogeneity of recruitment, extraneous movement, pH drop, limited O_2_ supply.	Section 4, esp. 4.3, Table 5
Restricted blood supply, oxygenation effects	Choice of exercise regime e.g. dynamic rather than isometric. Consider concurrent measurement of haemodynamic parameters with complementary methods, e.g. NIRS, (interleaved) ^1^H MR quantifying perfusion, dMb, *T*_2_ * contrast; caveat: BOLD and pH-driven effects.	Section 3
Reporting in studies	Report all acquisition parameters and results (also of relevant intermediate steps) necessary to understand and replicate the acquisition and quantification protocol; include coil type and size, flip angle, *TR*, exercise type and duration.	Section 4, esp. 4.1, Table 3

## Footnotes

* ATP is the product of ADP phosphorylation, a process commonly, but more loosely, referred to as ATP synthesis. This is biochemically the reverse of ATP hydrolysis, although the enzymes and pathways involved are very different; note that although ATP hydrolysis is far from thermodynamic equilibrium (which is what drives metabolic and mechanical work), the creatine kinase reaction (which also interconverts ATP and ADP) is always close to equilibrium

† The term ‘exercise’, as used throughout this article, refers to a period of muscle work which in most ^31^P MRS protocols consists of a series of muscle contractions separated by relaxation phases; ‘recovery’ refers to the data-collection period after cessation of the exercise part of the protocol.

‡ That is, if signal-contributing tissue is either equally exercising and has identical oxidative capacity (*Q*_max_), or is not exercising at all, and thus is not contributing to the depleted PCr signal.

§ Any substantial change in total phosphate (or in the sum of the concentrations of the two major components, Pi and PCr, which change in a near-equimolar fashion in opposite directions during exercise and recovery) suggests signal loss or gain due e.g. to coil movement.

‖ This can be thought of as a special case of relative method, with tissue water as the reference ‘metabolite’.

## Abbreviations used

ADP: adenosine diphosphate
ASL: arterial spin labelling
BOLD: blood oxygenation level dependent
CK: creatine kinase
FASTMAP: fast, automatic shimming technique by mapping along projections
FOG: fast-twitch oxidative glycolytic
FG: fast-twitch glycolytic
Δ*G*_ATP_: Gibbs free energy of ATP hydrolysis
GPC: glycero-3-phosphocholine
GPE: glycero-3-phosphoethanolamine
GAPDH: glyceraldehyde-3-phosphate dehydrogenase
Hb: haemoglobin
ISIS: image selected in vivo spectroscopy
IT: inversion transfer
*k*_PCr_: rate constant of post-exercise PCr recovery
LASER: localisation by adiabatic selective refocusing
MRSI: magnetic resonance spectroscopic imaging
MVC: maximum voluntary contraction force
Mb: myoglobin
NAD(P)H: 1,4-Dihydronicotinamide-adenine dinucleotide (phosphate), the reduced form of NAD(P)^+^
NIRS: near infrared spectroscopy
NOE: nuclear Overhauser effect
PDE: phosphodiesters
PGK: phosphoglycerate kinase
PME: phosphomonoesters
*Q*_max_: maximum rate of oxidative ATP synthesis or ADP phosphorylation (‘mitochondrial capacity’)
RF: radio frequency
SEM: standard error of the mean
SNR: signal-to-noise ratio
ST: saturation transfer
*τ*_PCr_: time constant of post-exercise PCr recovery
TCr: total creatine
SO: slow-twitch oxidative
VOI: volume of interest
*V*_PCr_: initial post-exercise PCr recovery rate
