# Supplementary material for: 31P magnetic resonance spectroscopy in skeletal muscle: Experts' consensus recommendations
Source: NMR Biomed. Author manuscript; Available in PMC 2021 Sep 24. (PMC8243949; doi:10.1002/nbm.4246)
Supplement: Appendix A [file EMS86008-supplement-Appendix_A.pdf]

APPENDIX A

In addition to the co-authors of this article, the following researchers, who constitute the Experts' Working Group on ^{31}P MR Spectroscopy of Skeletal Muscle, support the consensus paper and the recommendations therein.

Céline Baligand, CEA, MIRCen, Fontenay-aux-Roses, France; Pierre G. Carlier, NMR Laboratory, Neuromuscular Investigation Center, Institute of Myology AIM-CEA, Paris, France; Benjamin Chatel, Aix-Marseille University, CNRS, CRMBM, Marseille, France; Bruce Damon, Vanderbilt University, Nashville, Tennessee, USA; Linda Heskamp, Institute of Cellular Medicine, Newcastle University, Newcastle, UK; Milan Hájek, Institute for Clinical and Experimental Medicine, Prague, Czech Republic; Melissa Jooijmans, Amsterdam UMC, University of Amsterdam, The Netherlands; Martin Krssak, Medical University of Vienna, Austria; Juergen Reichenbach, IDIR, Universitätsklinikum Jena, Germany; Albrecht Schmid, Medical University of Vienna, Austria; Jill Slade, Department of Radiology, Michigan State University, East Lansing, USA; Krista Vandenborne, Department of Physical Therapy, University of Florida, Gainesville, Florida, USA; Glenn A Walter, Department of Physiology and Therapeutics, University of Florida, Gainesville, Florida, USA; David Willis, Norwich Medical School, University of East Anglia, Norwich, UK.
